# The Role of Immune Defects and Colonization of *Staphylococcus aureus* in the Pathogenesis of Atopic Dermatitis

**DOI:** 10.1155/2018/1956403

**Published:** 2018-05-02

**Authors:** Danuta Nowicka, Ewelina Grywalska

**Affiliations:** ^1^Department of Dermatology, Venereology, and Allergology, Wroclaw Medical University, Wrocław, Poland; ^2^Department of Clinical Immunology and Immunotherapy, Medical University of Lublin, Lublin, Poland

## Abstract

Atopic dermatitis (AD) is a condition with a complex and not fully understood etiology. In patients with AD, acute skin lesions are colonized by a greater number of *Staphylococcus aureus* (*S. aureus*) bacteria than chronic lesions, clinically unchanged atopic skin, or the skin of healthy people. Mechanisms promoting skin colonization by *S. aureus* include complex interactions among several factors. Apart from increased adhesion of *S. aureus* in atopic skin, defects of the innate immune response resulting in the lack of restriction of the growth of microorganisms also contribute to susceptibility to colonization by and infection with *S. aureus*. A deficiency in the endogenous antimicrobial peptides may be partly responsible for the susceptibility to colonization by and skin infection with *S. aureus* in patients with AD. Majority of isolated *S. aureus* stains are able to produce exotoxins, which act as superantigens. Moreover, anti-*S. aureus*-specific IgE was identified and measured in patients with AD, revealing that its level corresponds to the severity of the disease. This review of the literature attempts to identify factors that are involved in the pathogenesis of AD-related *S. aureus* skin colonization. In the light of presented mechanisms, a reduction of colonization may become both causative and symptomatic treatment in AD.

## 1. Introduction

Atopic dermatitis (AD) is a condition with a complex and, up till now, not fully understood etiology. The first reports of the disease date back to ancient times; however, the first reports in the literature and its presence in medical practice dates back to 1808 when Wilian made his pioneer “clinical” description of prurigo and of a prurigo-like condition with the special emphasis on the itchiness which is a characteristic for atopic dermatitis [1]. After many years of observation and experience, the term atopic dermatitis was introduced and described only in 1933 by Fred Wise and Marion Sulzberger [2, 3].

Despite the fact that many descriptions of the symptoms of AD and the causes of their occurrence in the course of the disease exist in the current literature, the entire picture of the disease is not fully elucidated. Possible causes of AD encompass disorders of the structure and function of the epidermal barrier, immune factors, and allergens as well as environmental and genetic factors [4]. The role of the contribution of infections, especially those caused by *Staphylococcus aureus* (*S. aureus*), seems to be very interesting and is regarded as important by some authors as well [5–7]. In the light of the latest reports, colonization of microorganisms can be considered as both one of the causative factors and a consequence of the disorders in atopic skin [8].

AD is a chronic disease with a variable course. The first skin symptoms appear usually during the first 3 months of life. Over the half of the cases (60%) is diagnosed before the end of the first year of life, and 90% before the end of the fifth year of life. Among adult patients, prevalence is higher in women than in men. The type of symptoms depends on the phase of the disease. The most common classification of the disease phases include infant, childhood, adolescent, and adult phase. In the most severe cases, skin changes are generalized. Itchy skin is the main symptom which appears in every type of AD and forces patients to scratch [9–11].

## 2. The Role of Epidermal Barrier

In AD, defects in skin barrier structure as well as impairment in functional integrity and reduced ability for self-renewal seem to play a role in releasing both an immune response and nonspecific inflammatory reaction [12, 13]. Increasingly, skin barrier defects are mentioned as one of the factors facilitating bacterial colonization. Skin barrier minimizes water loss from the epidermis and deeper parts of the skin as well as protects against environmental factors such as warmth or cold, penetration of potentially harmful substances, and colonization of pathological bacteria. The good condition of the epidermal barrier assures a healthy appearance and the proper functioning of the skin. In many diseases, the structure and, subsequently to it, the functioning of the epidermal barrier become altered.

The epidermal barrier is composed of corneocytes, which are the cells of the stratum corneum, lipids, and the natural moisturizing factor (NMF), which is produced during maturation of corneocytes. Corneocytes are formed during the process of maturation of keratinocytes and their migration from the basal layer of the epidermis toward the external surface of the skin. These cells are “dead,” flattened, and anucleated. The interior of the cells is filled with protein keratin [14]. During the migration from the basal layer, they lose the cell nucleus. Also, the expression of superficial proteins changes from keratin 5 and 14 to 1 and 2e as well as keratin 10. Keratin fibers are bound by filaggrin, which is the second in terms of the amount protein of the uppermost layers of the epidermis [15].

Currently, mutations of gene coding filaggrin are considered as one of the most important risk factors for AD, food allergies, and bronchial asthma [16–18]. In the terminal differentiation of keratin, a so-called cornified envelope (CE) is created which is a protein envelope responsible for epidermal barrier resistance to lytic enzymes [19]. This envelope is composed of filaggrin, loricrin, trichohyalin, involucrin, and intermediate keratin filaments among others. During differentiation of filaggrin, free amino acids and other substances that form NMF are released. NMF is a structure responsible for the absorption and binding of water in the protective layer of the epidermis. It is composed primarily of free amino acids, including salts of pyroglutamic acid, urea, and inorganic salts. NMF constitutes about 20% of the stratum corneum of the epidermis, but its proper functioning is guaranteed by the proper qualitative and quantitative composition. During differentiation of keratinocytes, synthesis of lipids of the extracellular matrix takes place and includes primarily ceramides, free fatty acids, cholesterol, and their esters. Ceramides constitute the major part of the extracellular matrix, which can contain even 40% to 50% of them [14, 20].

Another important component of the stratum corneum is cholesterol. The extracellular matrix contains about 25% of cholesterol. Cells of the basal layer are able to absorb cholesterol from circulation; however, it is almost entirely produced in the epidermis [21, 22]. The change in the composition of lipids and fatty acids in the skin of patients suffering from AD as well as the reduction of the amount of ceramides in the stratum corneum of the epidermis along with the increase in the amount of cholesterol seems to play the greatest role in the bacterial colonization. Heczko et al. showed that a shortage of medium-chain-length fatty acids may foster colonization by *S. aureus* in the epidermis, as they observed an inhibitory effect of the elevated concentration of capric, caprylic, and lauric acid on the growth of *S. aureus* [23].

## 3. Inflammatory Process

**Figure 1:**
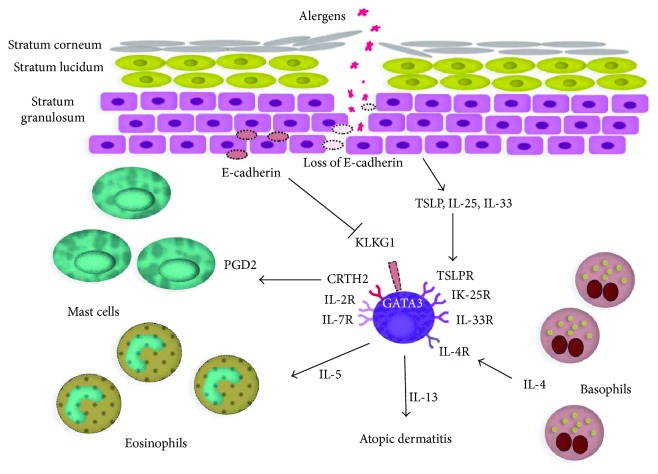
Presentation of mutual associations among epidermal barrier damage and immune dysregulations in atopic dermatitis.

Epidermal barrier damage by an inflammatory process facilitates colonization by microorganisms. Mechanical damage by scratching, environmental factors, and contact with detergents contribute to bacterial colonization as well. In the atopic skin, elevated pH is observed on the surface of the epidermis. Its value often reaches up to 8.00 [24, 25]. The structure of the epidermis along with presentation of mutual associations among epidermal barrier damage and immune dysregulations in atopic dermatitis is presented in Figure 1.

Among immune dysregulations observed in AD, an increase in the level of immunoglobulin E (IgE) and specific antibodies against various allergens plays a major role and seems to have the greatest clinical importance. Aeroallergens and food allergens predominate. An equally important mechanism of immune dysregulations seems to be a change in the cytokine profile synthetized by subpopulations of Th1 and Th2. A shortage in the Th1 subpopulation observed in those patients is associated with reduced secretion of interferon (IFN) gamma which in turn may reduce the activity of natural killer (NK) cells. Reduced production of IFN gamma is also associated with a decrease in the number of circulating T lymphocytes and translates into an elevated ratio of CD4+/CD8+ lymphocytes. Identical results are obtained both from peripheral blood and skin lesions. The proliferative response of lymphocytes after stimulation by anti-CD3 antibody (OKT-3) and mitogens decreases as well [26, 27].

Another disorder of immune response observed in AD is a dysregulation of granulocytes. They mainly encompass chemotaxis and a capacity for generation of free radicals. In the skin, the increase in the number of mast cells which are responsible for secretion of neurotransmitters is observed. The pathways of activation of complements seem to be very interesting in patients with AD. The main factor which contributes to its elevated activity seems to be the IgE-anti-IgE complex. The elevated concentration of IgE may also play a role in the increased susceptibility to infections [13, 28].

## 4. *S. aureus* in Atopic Dermatitis

In patients with AD, acute skin lesions are colonized by a greater number of *S. aureus* bacteria than chronic lesions, clinically unchanged atopic skin, or the skin of healthy people. Mechanisms promoting skin colonization by *S. aureus* include complex interactions among several factors. They encompass the dysfunction of the skin barrier, an elevated synthesis of *S. aureus* adhesion molecules in the extracellular matrix, decreased lipid content in the skin, changes in the pH of the skin surface in the direction of alkalinity, and defective innate immune responses due to decreased production of endogenous antimicrobial peptides [4, 12, 28, 29].

## 5. Increased Adhesion of *S. aureus* to the Skin

*S. aureus* is able to form a biofilm composed of a hydrated matrix of polysaccharides and proteins, which facilitates cell adhesion [30]. Adhesion of *S. aureus* takes place mainly in the stratum corneum in the epidermis, and it is mediated by fibronectin and fibrinogen. It was shown that adhesion of *S. aureus* to the surface of the skin is increased in patients with AD in comparison to healthy people. In AD, the inflammatory process is caused by allergens and leads to a damage of the skin barrier and as a result, to the exposure of the extracellular matrix to *S. aureus*. The extracellular matrix adhesins of the *S. aureus* cell membrane include dermal and epidermal fibronectin and laminin which become exposed in the skin with lesions and, in this way, increase adhesion of *S. aureus*. Scratching also increases binding of *S. aureus* cells though disturbances in the skin barrier and release of cytokines which regulate expression of extracellular matrix adhesins for *S. aureus*. Additionally, the skin of AD patients shows an increased deposition of fibronectin in the stratum corneum. This factor may increase *S. aureus* binding to the skin.

In the murine model, it was shown that *S. aureus* binding to the skin was significantly higher in the case of skin lesions with Th2 cell-mediated inflammation than in inflammatory lesions caused by a predominance of Th1. Moreover, an increased *S. aureus* binding was absent in mice with an interleukin- (IL-) 4 gene knockout suggesting that IL-4 plays an important role in the mechanism of *S. aureus* binding to the atopic skin. On the contrary, the increase in *S. aureus* binding was observed only in the skin of a healthy mouse which was incubated *in vitro* with IL-4, but not with IFN gamma. Therefore, it is suggested that IL-4—a crucial cytokine of Th2—induces production of staphylococcal adhesin and fibronectin by skin fibroblasts. A protein that binds fibronectin with *S. aureus* has a dual function as it also binds with fibrinogen. It has been shown that in the murine model, *S. aureus* mutants with a selective deficiency in proteins binding fibronectin or fibrinogen presented with lower adhesion to allergy-sensitive Th2 cells, but not Th1, in comparison with wild-type strains [31, 32].

Similarly in human studies, *S. aureus* mutants presenting a selective deficiency in fibronectin-binding proteins and fibrinogen-binding proteins exerted reduced binding to the AD skin in comparison with the psoriatic skin and the skin of healthy people. On the contrary, *S. aureus* mutants presenting with a selective deficiency in adhesins to collagen did not exert reduced binding to Th2 cell-mediated inflammatory skin lesions. Additionally, when *S. aureus* was preincubated with human serum albumin and either fibronectin, fibrinogen, or collagen in order to block specific binding-proteins of *S. aureus*, only fibronectin and fibrinogen significantly suppressed *S. aureus* binding. Therefore, those findings suggest that fibronectin and fibrinogen, but not collagen, contribute to the *S. aureus* binding to the skin with Th2 cell-mediated inflammatory skin lesions [33]. It is known that IL-4 is a crucial cytokine secreted by Th2. It induces synthesis of fibronectin by skin fibroblasts. Fibronectin along with fibrinogen released from the plasma during the acute phase of the disease enables *S. aureus* to bind to the skin. Thus, it seems that blockage of *S. aureus* binding to fibrinogen and fibronectin may be a therapeutic target reducing colonization by *S. aureus* in patients with AD [31, 32].

The change in lipid composition of the stratum corneum of the epidermis is another factor contributing to the increased colonization by *S. aureus* in the skin of patients with AD. A decreased level of ceramides is the primary defect of differentiation of the epidermis in patients with AD compared to healthy people [34, 35]. Ceramides are the main molecules that help in maintaining water retention and serve as the main adhesive molecules for structural proteins of the extracellular matrix of the stratum corneum of the epidermis. A decrease in ceramides may lead to increased transepidermal water loss and contribute to a dry and flaky appearance. Such skin is susceptible to *S. aureus* colonization [36, 37]. It was reported that in atopic skin, *S. aureus* stimulates the hydrolysis of ceramides itself using bacterial ceramidase [38].

Sphingosine is an important lipid component of the skin as well. In normal conditions, it exerts a strong antimicrobial effect against *S. aureus*. Nevertheless, patients with AD present with a reduced concentration of sphingosine in the stratum corneum as a result of a reduced activity of ceramidase acid and a reduced level of ceramides. These factors are conducive to *S. aureus* colonization. Changes in pH on the surface of the epidermis observed in atopic skin seem to be another factor which favors *S. aureus* colonization. In a study conducted years ago, the authors reported that an optimal basicity for adhesion of *S. aureus* to corneocytes ranges between 7 and 8 [39].

## 6. Defects of Innate Immune Response

Apart from increased adhesion of *S. aureus* in atopic skin, defects of the innate immune response resulting most of all in the lack of restriction of the growth of microorganisms also contribute to susceptibility to colonization by and infection with *S. aureus*. Comparison between AD (Th2-mediated inflammatory disease) and psoriasis (Th1-mediated inflammation) showed that about 30% of patients with AD suffered from skin infections, while only 6.7% patients with psoriasis had skin infection despite impaired skin barrier functioning in both groups of patients [40]. This discovery suggests that inflammatory lesions in the skin caused by Th2, but not by Th1, may be associated with defects of the innate immune response.

There are two main classes of endogenous antimicrobial peptides in the human skin: beta-defensins and cathelicidins [41]. These antimicrobial peptides are produced by keratinocytes and act against bacteria, viruses, and fungi. One of the proposed mechanisms of action explains antimicrobial action by the possibility of a disruption of the cell membrane in order to impair intracellular functions [42]. Some of these endogenous antimicrobial peptides (e.g., human beta-defensin 1 (HBD-1)) are produced constitutively, while expression of other antimicrobial peptides (e.g., human beta-defensin 2 (HBD-2), cathelicidin LL-37) is induced by tumor necrosis factor *α* (TNF-*α*) after the development of skin inflammation or mechanical injury [43, 44].

Animal models revealed that endogenous antimicrobial peptides are essential for defense against bacterial infection in the skin. What is more, a combination of HBD-2 and LL-37 exerts synergistic antimicrobial action which is greater than that exerted by one antimicrobial peptide alone. Thus, the expression of both antimicrobial peptides is important for the innate immune response of the skin. In a recent study, the expression of endogenous antimicrobial peptides HBD-2 and LL-37 was compared among skin lesion in AD and psoriasis as well as healthy skin. The expression of examined peptides was determined by immunohistochemical staining and analyzed by immunodot blot analysis (for LL-37) and Western blot analysis (for HBD-2). Real-time reverse-transcriptase-polymerase-chain-reaction (RT-PCR) was used to confirm the relative expression of both peptide mRNAs. Results of the study showed that expression of HBD-2 and LL-37 was lower in lesions from patients with atopic dermatitis in comparison to those from psoriatic patients and healthy people [45]. Therefore, a deficiency in the endogenous antimicrobial peptides may be partly responsible for the susceptibility to colonization by and skin infection with *S. aureus* in patients with atopic skin. After getting access to the skin, colonies of *S. aureus* grow uncontrollably because of the shortage of antimicrobial peptides. According to another study, a shortage of antimicrobial peptides may be rather associated with Th2-mediated inflammation than with Th1-mediated inflammation. Additionally, among Th2 cytokines, IL-4 alone or together with IL-13 significantly downregulates, induced by TNF-*α*, expression of HBD-2 in human keratinocytes. Taking above into consideration, the data suggest that reduced expression of endogenous antimicrobial peptides in AD is a result of Th2 immune response [46, 47].

## 7. Superantigens

Superantigens are a group of bacterial and viral peptides recognized for their ability to stimulate a large number of various clones of T cells to produce cytokines [48]. After processing and presentation by antigen-presenting cells via molecules of major histocompatibility complex (MHC) class II, traditional peptide antigens recognize and bind to those of T cells which have 5 specific variable elements (V*β*, D*β*, J*β*, V*α*, and J*α*) within T cell receptors (TCR). So the amount of T lymphocytes activated with conventional peptide antigens accounts for approximately 0.01 to 0.1% of the total T lymphocyte population. In contrast to conventional peptide antigens, superantigens do not require processing and antigen presentation by antigen-presenting cells. Superantigens bind directly to the variable *β*-domain of the *β* chain (V*β*) of the TCR molecule (TCRV*β*) and the MHC class II on the surface of antigen-presenting cells outside the groove binding the peptic antigen of the MHC. They recognize and stimulate T lymphocytes with specific TCRV*β* domains, which results in activation of huge amounts of polyclonal T cells—up to 15–20% of the total population of T lymphocytes, hence the term “superantigen” [49].

In over 90% of patients with AD, colonization by *S. aureus* was detected on the surface of the epidermis. Over 70% of isolated *S. aureus* stains are able to produce exotoxins, including staphylococcal enterotoxins A, B, and C (SEA, SEB, and SEC) as well as toxic shock syndrome toxin-1 (TSST-1) [50, 51]. These exotoxins act as superantigens. They penetrate the epidermal barrier and exacerbate the course of inflammation. Many current studies emphasize the association between colonization by *S. aureus* and the severity of AD [52, 53].

## 8. *S. aureus* as Allergen

Skin colonization by *S. aureus* in the course of AD, as a cause of an overreaction of the immune system to the presence of those bacteria, exerts a toxic effect on keratinocytes, stimulates lymphocytes to secrete IFN, and as a consequence, leads to the development of a chronic type of the disease. The bacteria itself and their metabolites induce activation of T lymphocytes, macrophages, and antigen-presenting cells which lead to increased production of IgE and IgG among others. The elevated level of IgE is one of the characteristic symptoms of the immune response to allergen. Anti-*S. aureus*-specific IgE was identified and measured in patients with AD. Its level corresponds to the severity of the disease [54, 55].

## 9. Therapeutic Implications

Many mechanisms facilitate *S. aureus* colonization on the surface of the epidermis, and simultaneously, many processes induced by those microorganisms exacerbate the course of the disease. Thus, *S. aureus* colonization is both the cause and consequence of the disease. This condition translates into treatment of AD and a major role for both topical and systemic antibiotics. Unfortunately, more and more often, *S. aureus* becomes resistant to the most commonly used preparations. In the study conducted by Bessa et al., the frequency of fusidic acid and mupirocin resistant strains was low; however, the high rate of neomycin and bacitracin resistance is alarming as those antibiotics are common in clinical practice [56].

Considering the mechanisms of *S. aureus* colonization in the atopic skin, it seems reasonable to reduce the use of antibiotics for nonspecifically or indirectly acting substances that limit the growth of these bacteria. Broadly used emollients can help in restoring the composition of the epidermal barrier. Additionally, probiotics which gain in popularity as an ingredient of topical preparations seem to be a natural and very promising weapon that inhibits growth of pathogenic *S. aureus* [57]. In the light of presented mechanisms, a reduction of colonization may become both causative and symptomatic treatment in AD.
